# Death from Laudanum in Three-quarters of an Hour

**Published:** 1854-11

**Authors:** 


Death from Laudanum in three-quarters of an hour.—The following account of a case, which occurred in 1849, and was then reported, verbally only, to the Society, has been furnished, at the request of the Secretary, by Dr. G. H. Lyman. Mrs. ——; aged fifty-two years. Intemperate; has been drinking more or less for three weeks. An ounce of laudanum was taken by the patient, with suicidal intent. Dr. L. saw her in fifteen minutes. She said she had taken the laudanum for diarrhœa; was able to walk about with little or no assistance; begged to be allowed to lie down.

Nothing peculiar in pulse, as to force or frequency; pupils natural; external temperature natural; no evidence of sopor. Dr. L. gave two emetics of sulph. zinc.; could get no stomach pump. No effect produced by emetics.

In thirty-five minutes after taking the laudanum, she began very suddenly to lose her pulse and muscular power; slight spasms were observed; the lips became livid; there was a spasmodic dropping of the lower jaw; the extremities were cold, and, in ten minutes, she was unmistakably dead. The moment her pulse began to fail, frictions and sinapisms were resorted to, and preparation was made for the douche and artificial respiration; but she died before they could be instituted.

She had taken a hearty breakfast about three hours before. Her husband says her habit of drinking was induced by taking great quantities of tinctures (that of opium among others), after an attack of traumatic tetanus, ten years previous to her death.

Three quarters of an hour only elapsed from the ingestion of the laudanum to her death. Beck reports a case fatal in two hours. Christison says, seven to twelve hours is the average duration.

A case is reported in the *Boston Medical and Surgical Journal*, vol. xi, p. 285, fatal in two and a half hours. Christison also remarks that intoxication retards the action of opium.—*American Journal of Medical Sciences.*

